# Anatomy of meat cuts: integrating 3D scanning and virtual reality in veterinary education and training

**DOI:** 10.3389/fvets.2025.1680785

**Published:** 2025-10-23

**Authors:** Nedžad Hadžiomerović, Kenan Čaklovica, Nejra Dučić, Migena Gjoni Gündemir, Anel Vejzović, Neira Fazlović, Rizah Avdić, Faruk Čaklovica, Faruk Tandir

**Affiliations:** ^1^Department of Basic Sciences of Veterinary Medicine, University of Sarajevo-Veterinary Faculty, Sarajevo, Bosnia and Herzegovina; ^2^Department of Food Safety and Environmental Protection, University of Sarajevo-Veterinary Faculty, Sarajevo, Bosnia and Herzegovina; ^3^Institute of Graduate Studies, İstanbul University-Cerrahpaşa, İstanbul, Türkiye

**Keywords:** digital tools, immersive technology, interactive learning, muscle structure, skill development

## Abstract

Understanding meat categorization is a fundamental component of veterinary education, especially within the context of food hygiene and public health. Veterinary students must grasp legal classifications of meat, which depend on variables such as species, age, quality, and processing techniques. This knowledge is essential for accurate meat inspection, labeling, and compliance with both national and international food safety standards. Despite prior exposure to muscle anatomy in anatomy course, students often face challenges in applying this knowledge to practical meat classification tasks. This study aimed to assess the effectiveness of three distinct instructional methods in improving veterinary students’ ability to identify meat categories and associated muscle structures: traditional classroom teaching, computer-based instruction using 3D models, and immersive virtual reality (VR). Participants included fourth-year veterinary students during the summer semester of the 2024/2025 academic year. To facilitate digital learning, a dedicated 3D model library “3DMeat” was developed as well as virtual reality environment. Results indicate that technology-enhanced instructional approaches, can significantly enhance student engagement and understanding of complex topics such as meat categorization. Initial test scores were highest in the group using 3D models (16.3 ± 4.1), followed by the traditional lecture-based group (15.6 ± 3.07), and the VR group (11.7 ± 5.1). However, a follow-up assessment conducted 2 weeks later revealed that VR group demonstrated the highest retention of knowledge. These findings suggest that although immediate performance may vary, immersive learning environments such as VR can foster stronger medium-term retention of complex material.

## Introduction

Graduates of the veterinary medicine program attain the “Day One Competences” indicating that they possess the essential knowledge, skills and professional attributes required to independently perform tasks and duties of the veterinary profession. These competences include the understanding, principles and skills related to food safety and quality, veterinary public health and One Health concept (1). Veterinary medicine plays a critical role in the prevention and control of the transmission of foodborne pathogens throughout the food chain (2). At Veterinary Faculty of University of Sarajevo, during practical course “Hygiene and Technology of Meat and Meat Products” students learn about post-mortem inspection of food-producing animals and accurately identify conditions that affect the quality and health safety of products of animal origin. With the growing interest in meat authenticity, methods for meat authentication are commonly classified according to areas most susceptible to fraud: meat origin, meat substitution, meat processing treatment and non-meat ingredient addition (3). The rising incidence of meat fraud represents a global challenge, posing significant public health risks and commercial concerns (4). One of the persistent challenges in veterinary meat education is the accurate identification of individual muscles within meat cuts. This difficulty is partly due to the temporal gap between anatomy courses—typically taught in the first semester of veterinary programs—and meat science courses, which are usually scheduled in subsequent semesters (e.g., the eighth). As a result, students often struggle to recognize specific muscles within commercial meat cuts. This highlights the importance of enhancing veterinary education on meat anatomy, with a focus on muscle identification, potential mislabeling, and the structural composition of meat cuts.

The use of different distant learning methods in veterinary medicine has become more popular since the COVID-19 pandemic (5). Technologies like augmented reality (AR), virtual reality (VR), 3D models, video lectures, and other online tools support 3D visualization of different structures, making learning more interactive for students (6). The utilization of virtual reality (VR) provides an immersive experience in a 3D environment (7). VR offers varying levels of interactivity through the use of equipment like headsets or gloves (8). It consists of three basic ideas: immersion, interaction and involvement. Its interactive feature allows the user to manipulate virtual objects (9). Several studies in which VR was used in educational purposes have shown improved knowledge retention, clinical reasoning and student satisfaction (10, 11). Additionally, it was proven that immersive VR improves students’ academic achievement, as well as decreases their cognitive load (12). Another potential benefit of using simulators in veterinary training is their ability to lower students’ anxiety and stress when confronting real-life clinical situations (13, 14). According to the study conducted by Arif (15), increased exposure and more practice with VR can significantly improve students’ learning experience.

VR technology in veterinary medicine has mostly been used in teaching anatomy (5, 16), simulating various medical procedures (17, 18) and simulating abattoir visits (11, 19). However, the application of innovative digital tools for the identification of different meat cuts, particularly those providing detailed anatomical descriptions, remains limited, especially within virtual reality (VR) environments.

Based on previous studies published on the use of 3D models and virtual reality in medical and veterinary education, which highlight positive student feedback and better results, we hypothesize that students using 3D models and VR will achieve test scores comparable to or higher than those achieved through traditional classroom instruction.

## Materials and methods

### 3D scanning of the meat cuts

**Figure 1:**
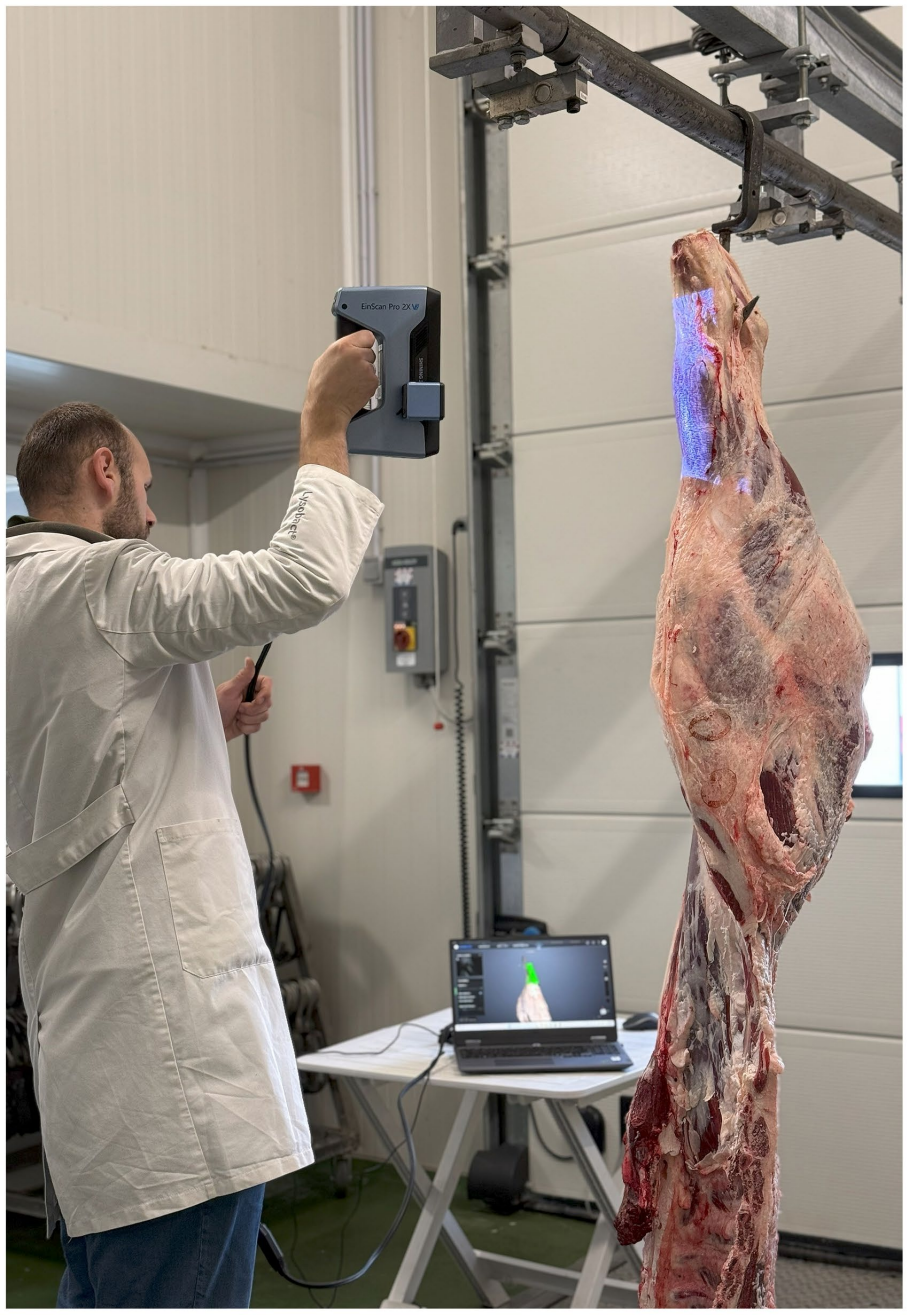
The process of 3D meat scanning.

For the 3D scanning of the meat cuts, Einscan Pro 2X (Shining 3D) scanner was used. This multifunctional handheld 3D scanner enables creating full-color texture along with geometry. The 3D scanning process was conducted in collaboration with a local meat industry facility, with strict adherence to meat hygiene protocols and temperature control (Figure 1). To ensure comprehensive capture of each meat cut’s geometry, the specimens were positioned as standard commercial meat portions and scanned from multiple angles and orientations. The post processing was performed with the software EXModel (Shining 3D) and all files were recorded as OBJ files.

### Virtual library—3DMeat

**Figure 2:**
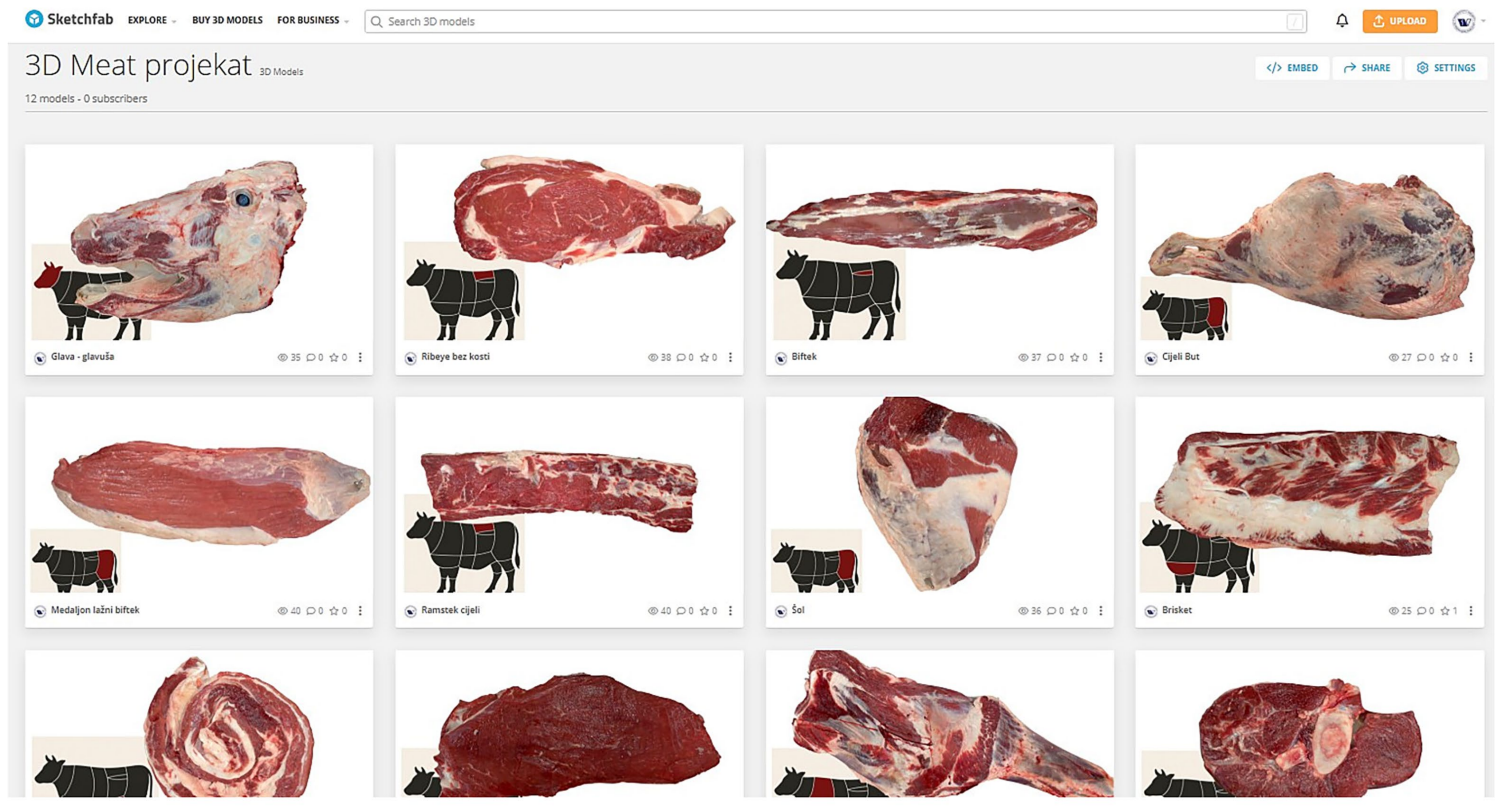
The 3D meat cuts collection on the Sketchfab platform.

The scanned files were uploaded to Sketchfab, one of the largest platforms for 3D model sharing, under the profile “University of Sarajevo-Veterinary Faculty.” A particular collection titled “3DMeat Project”^1^ was created for the research purpose and is publicly accessible. A total of 12 3D models were uploaded, each accompanied by descriptive metadata including the name of the meat cut, the constituent muscles and the corresponding meat category, along with a diagram of its anatomical location (Figure 2).

### Virtual reality environment

**Figure 3:**
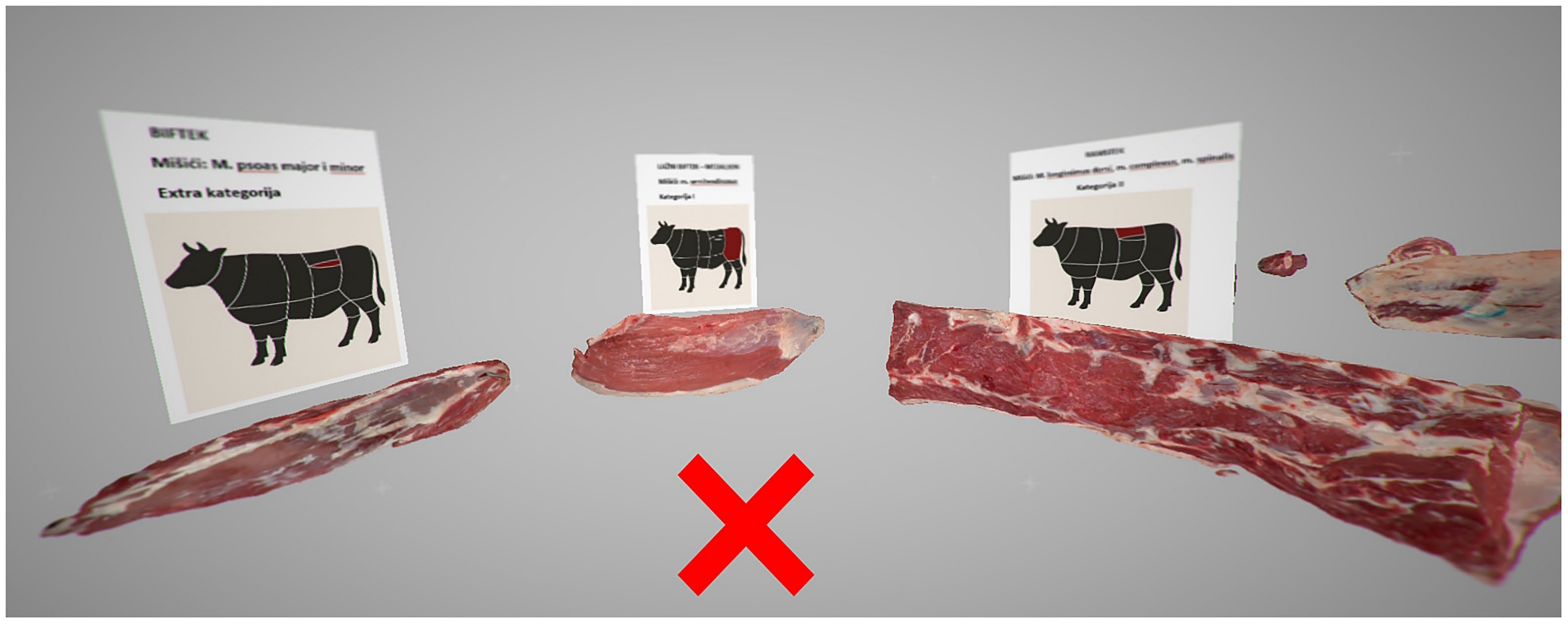
Image created in the virtual reality environment. The X represents the position of the user. Three meat cuts with the image above displaying name of the cut, the muscles, corresponding meat categories, and anatomical location based on a schematic representation of cattle.

To develop virtual reality (VR) environment, we utilized the Open Brush application, a free VR tool originally designed for three-dimensional painting within immersive virtual spaces. The application was operated using the HTC Vive Pro 2 headset (HTC Corporation, Taiwan), enabling interactive engagement with the 3D content in a fully immersive setting. The VR app was suitable for this purpose as it allows the user to import 3D models (3D scanned meat cuts) and images (anatomical location) in virtual environment. For the purposes of the study, two virtual stations were created, each featuring six distinct meat cuts. For every cut, a corresponding 3D model was imported into the VR environment, with an informational panel placed above each model displaying key details, including the name of the cut, the constituent muscles, corresponding meat categories, and its anatomical location (Figure 3).

**Figure 4:**
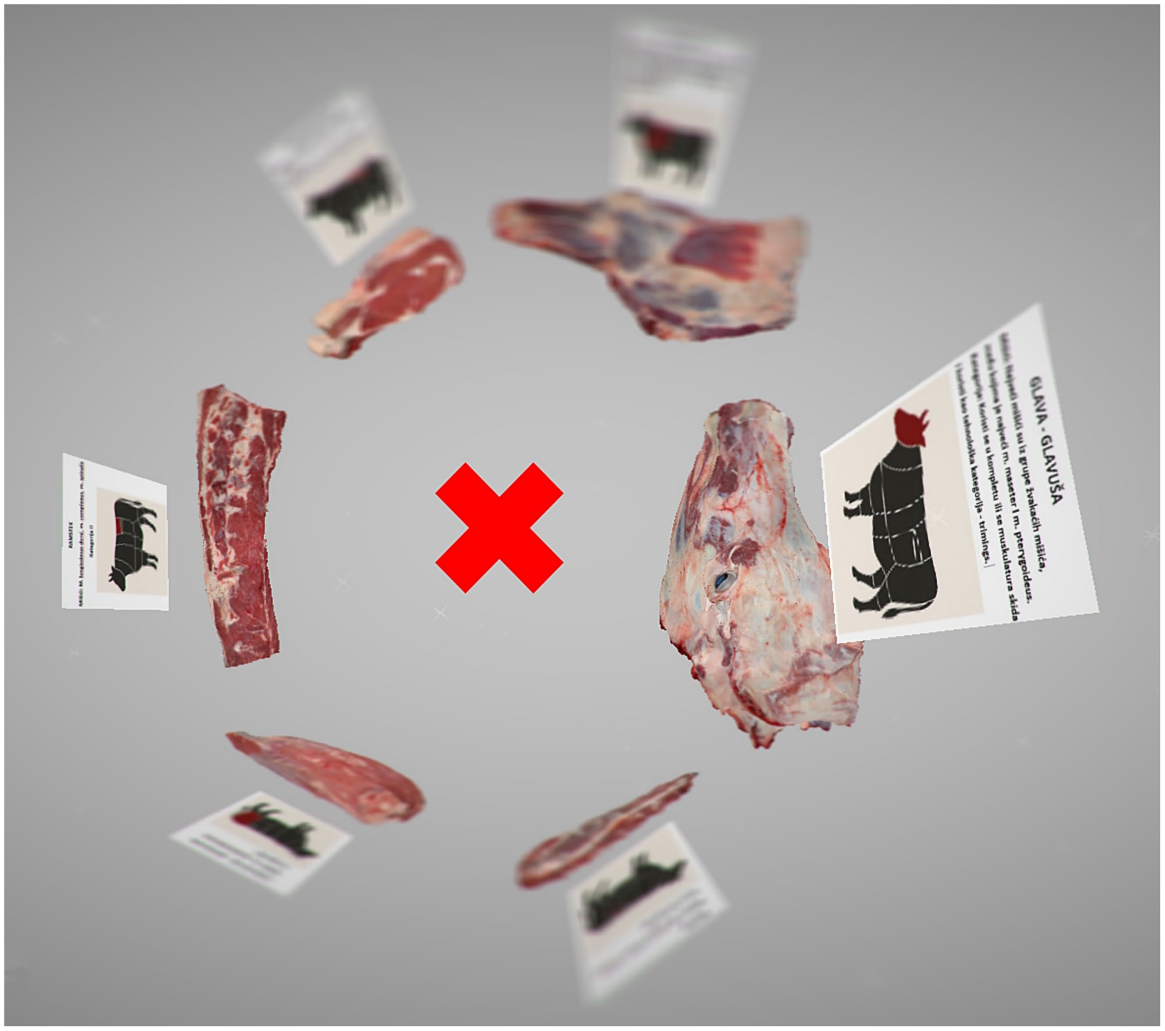
Image created in the virtual reality environment. The X represents the position of the user. Circular organization minimizes movement within the virtual space.

The six meat cuts were arranged in a circular configuration, allowing users to step into the center of the circle to observe and interact with each model from multiple angles (Figure 4). This organization was intentionally designed to minimize user movement within the virtual space, thereby reducing the risk of disorientation or discomfort, particularly among first-time VR users. Prior to the VR sessions, students were provided with instructions on the basic use of the controls, enabling them to manipulate the 3D objects and navigate within the virtual environment effectively.

### Study design

The research was conducted with fourth-year students in the integrated study program at the University of Sarajevo-Veterinary Faculty. The students were enrolled in the practical course “Hygiene and Technology of Meat and Meat Products” during the summer semester of the 2024/2025 academic year. A total of 25 students participated voluntarily in the study (68% female and 32% male), representing 60% of the overall student cohort. All participants provided informed consent. The students were randomly assigned to three groups, each corresponding to a different teaching approach: traditional classroom learning (control group; *n* = 8), computer-based instruction with 3D scanned models (*n* = 8), and immersive virtual reality (VR; *n* = 9). The duration of the lecture was 45 min for both the traditional and 3D models groups, while the individual VR sessions lasted approximately 10 min. The lecture content was developed collaboratively by the researchers and course lecturers. Twelve of the most important and recognizable meat cuts were selected for the research. For each cut, four key aspects were addressed: the name of the meat cut, the muscles comprising the cut, its classification within meat categories, and its anatomical location. The first group, which received traditional classroom instruction, attended a standard practical session delivered by the lecturers using a PowerPoint presentation. The 45-min lecture covered identical content on 12 meat cuts, including corresponding images and descriptive information. The second group engaged in self-directed learning using computer-based instruction with 3D models of the meat cuts. Over the course of 45 min, students independently manipulated the 3D models and reviewed the associated content. The third group experienced the content individually through a 10-min immersive VR session. While one student was engaged in the VR experience, the remaining students simultaneously reviewed the same material projected on a large screen, allowing them to continue learning during other participants’ sessions. Upon completion of the lecture or VR session, each group completed an identical questionnaire designed to assess their short-term knowledge of the various meat cuts presented during the instructional period. The questionnaire consisted of 20 multiple-choice questions, five of which were images of different meat cuts that students interacted with during the learning sessions (Supplementary material 1). Two weeks later, students completed a follow-up assessment using the same set of questions to evaluate their medium-term retention of the learning content. The questionnaire was developed and its content validity was established through review by subject-matter experts in the field.

### Statistical analysis

The SPSS package program was used for statistical analysis (SPSS for Windows, version 22.0). The descriptive statistics tool was applied to the obtained data to present the mean, median, standard deviation and range of the group results. Prior to statistical analysis, normality of data distribution was assessed by Shapiro- Wilk test of normality. If assumption of normality was met, Mixed - ANOVA was used to compare group scores with the significance level set at *p* < 0.05. Otherwise, Kruskal-Wallis test was used. To compare the groups more thoroughly, we included Cohen’s d effect size comparison of the groups. Retention Rate, Mixed-ANOVA with interaction and Paired t-tests were used to examine the pattern of variance across groups and to determine whether the retention after 15 days was different between groups.

## Results

**Figure 5:**
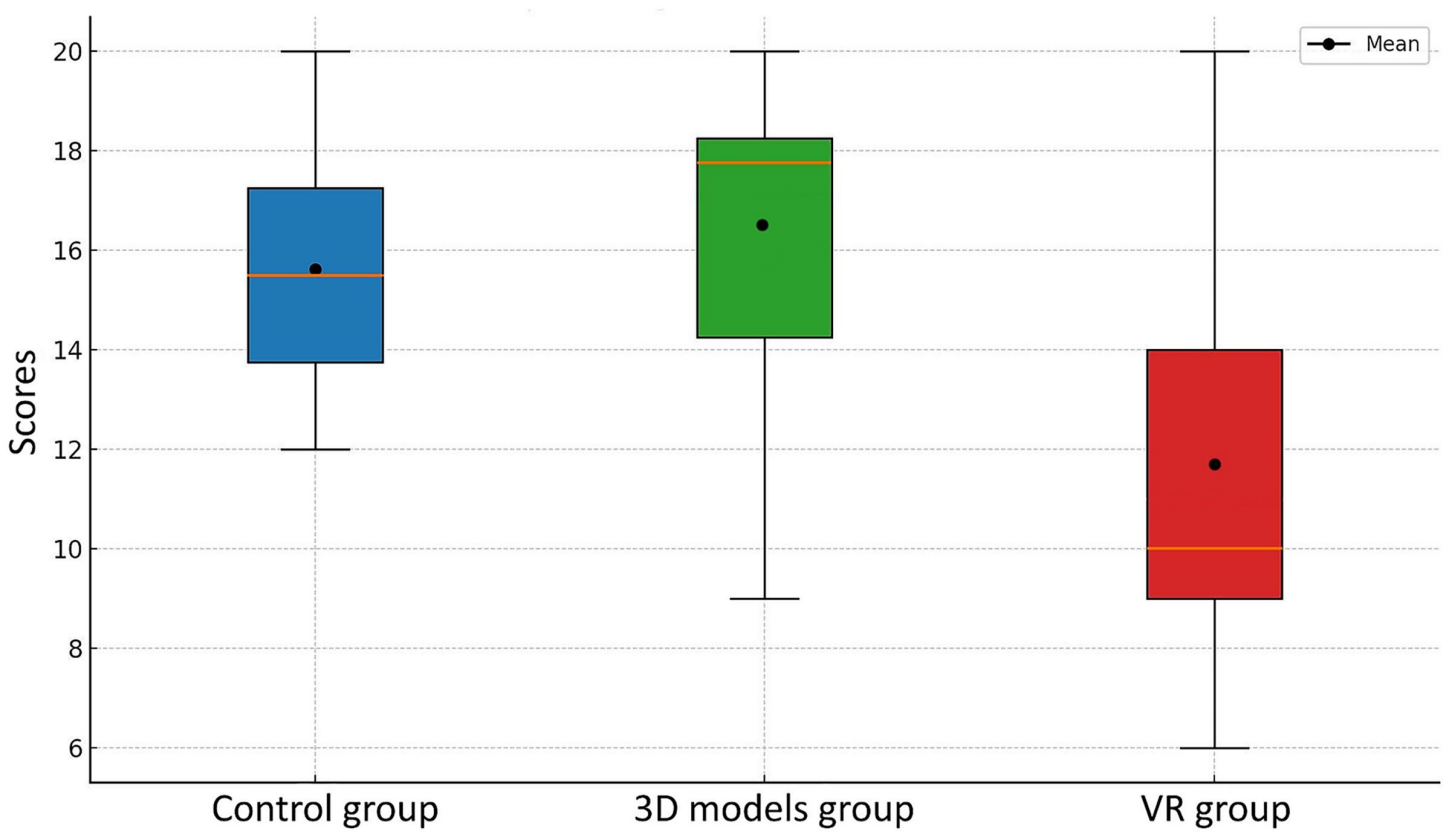
Student test scores across three experimental groups measured immediately after the learning session.

To test the hypothesis, exam scores from a 20-question test were compared across all three cohorts using a boxplot (Figure 5), accompanied by descriptive statistical analysis. The analysis includes measures of central tendency (mean, median), dispersion (standard deviation), and range. Results of Shapiro- Wilk test for all groups (control, 3D models, VR) concluded the data is normally distributed including the data of the follow-up assessment. The 3D models group achieved the highest overall performance, with a mean score of 16.38, 95% CI [12.92, 19.84]. The median was higher than the mean, suggesting a slight negative skew in the distribution. The traditional classroom (control) group ranked second (15.62, 95% CI [13.05, 18.19]), with closely aligned mean and median values, indicating a symmetrical distribution of results. In contrast, the VR group demonstrated lower scores (11.67, 95% CI [7.71, 15.63]) compared to the other groups. Additionally, this group showed the highest variability and the widest range in scores, which may reflect challenges in the effectiveness of the VR method or its insufficient adaptation to the students’ learning needs. Differences between the group scores were not significant, even though they were close to the borderline of statistical significance (*p* = 0.067). When comparing groups by Cohen’s d effect size, considerably large differences were present. The classroom group performed higher than the VR group (*d* = 0.92, large effect), 3D models group score was also higher than VR group (*d* = 1.01, large effect) whereas the difference between traditional classroom group and 3D models group was negligible (*d* = −0.21, small effect).

**Figure 6:**
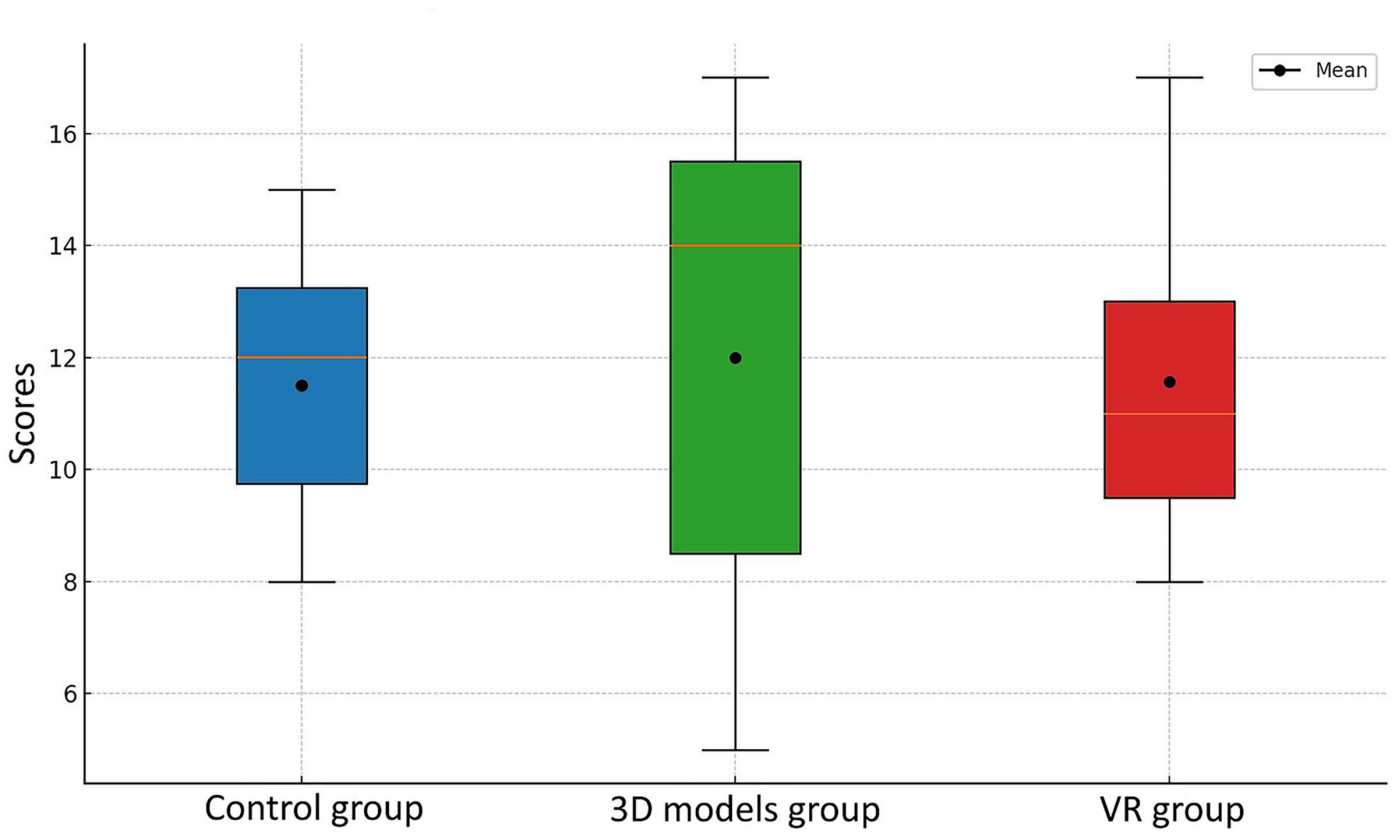
Student test scores across three experimental groups measured 2 weeks after the learning session.

Two weeks after the initial learning session, a follow-up assessment was conducted and a substantial decline in test scores was observed in both the control and 3D models groups (Figure 6). Despite achieving the highest mean score (12.85, 95% CI [9.42, 16.28]), the 3D models group exhibited a high standard deviation, suggesting considerable variability in individual performance. The control group demonstrated relatively stable outcomes (12.12, 95% CI [10.03, 14.21]), with moderate variability among participants. In contrast, the VR group maintained a performance level very similar to that of the initial test (11.57, 95% CI [8.98, 14.16]). Retention test scores did not differed significantly (*p* = 0.90) and their Cohen’s d effect size was small for all pairs (*d* = 0.19; *d* = 0.35; *d* = −0.21). The similarity between the mean and median values in this group indicates a symmetrical distribution and a consistent pattern of results. To compare the results between the first and second tests, the retention rate was calculated using the following formula:


$$\text{Retention rate}\;(\%)=[\frac{\text{Score after}\;2\;\text{weeks}}{\text{Score after session}}]\times 100$$

The VR group demonstrated the highest retention rate at 99.1%, indicating that students maintained nearly the same level of knowledge 2 weeks after the initial test. However, despite this high retention, their overall performance remained the lowest across both assessments. The 3D models group exhibited a retention rate of 78.5%, while the control group followed closely with 77.6%, suggesting a moderate decline in knowledge retention over time.

Results of performed Mixed-ANOVA showed there was no difference between test scores across groups regardless of test time. Main effect of the test was not statistically significant. In addition, the effect of interaction between Group x test was not significant [*F*(2, 21) = 2.58, *p* = 0.10] indicating that there is no significant difference in the pattern of change from test 1 to follow-up test across groups (Table 1). Pairwise t test, additionally conducted to assess whether the test scores differed at the level of every group, showed there is statistically significant difference in the results of Control group (Table 2).

**Table 1:** Summary of Mixed-ANOVA with interaction included.

Effect	DFn	DFd	SSn	SSd	*F*	*p*	*p* < 0.05	Ges
(Intercept)	1	21	8190.188	321.813	534.454	2.03E-16	*	0.921
Group	2	21	46.500	321.813	1.517	2.42E-01		0.062
Test	1	21	77.521	379.813	4.286	5.09E-02		0.099
Group: test	2	21	93.167	379.813	2.576	9.99E-02		0.117

**Table 2:** Results of paired *t*-test.

Group	t-statistic	*p*-value	Mean difference (test1–test2)
3D models	2.062	0.078	4.875
Control	2.855	0.025	4.125
VR	−0.567	0.589	−1.375

## Discussion

In the presented study, students’ ability to identify meat categories and describe muscle anatomy following instruction through three different teaching methods was investigated. This research was based on the hypothesis that students using 3D models and VR will achieve test scores comparable to or higher than those of students receiving traditional classroom instruction.

Analysis of the data reveals that the VR group demonstrated very similar results in both the initial and follow-up assessment with the highest retention rate (99.1%), even though their overall test scores were lower compared to the other two groups. On the other hand, the 3D models group demonstrated the highest overall scores. Numerous previous studies have explored the use of virtual reality (VR) in medical and veterinary education (11, 18, 19). The majority of these studies have focused on anatomy, recognizing it as a foundational component of medical training due to its highly visual and tactile nature. However, only a limited number of studies have provided detailed, step-by-step descriptions of the procedures required to create VR content in a simple, cost-effective, and accessible manner. The integration of photogrammetry for generating 3D models and VR-based anatomy learning modules within traditional cadaver-based anatomy courses has been described in terms of methodology, benefits, and associated challenges (20). Additionally, the study utilized affordable equipment like standard digital camera, computer, and software. Promising results have also been reported with the use of smartphone cameras and freely available software, making the process even more feasible (21). In our study, 3D scanning technology was employed to develop high-resolution, realistic 3D models for educational use. This technology has demonstrated significant effectiveness not only in enhancing anatomy education but also as a valuable tool in research. The exceptional precision and accuracy afforded by this technology are crucial for conducting detailed geometric studies in three dimensions (22–25).

To enhance learning outcomes, various teaching approaches were developed. A previous study on the integration of VR in medical education demonstrated a high level of student satisfaction, as well as improved knowledge and retention (10). In research conducted by Seguino et al. (19), students reported that the use of VR slaughterhouse simulator was particularly helpful for visual learners, as it supported the transition from theoretical understanding to practical implementation. Another study by Schirone et al. (26) showed that students preferred 3D models to 2D learning material as they enhanced their motivation. However, the differences between students who used 3D models and 2D images were not significant, suggesting that 3D scans do not necessarily lead to higher learning outcomes. Our study demonstrated that self-directed learning utilizing 3D models and supplementary content yielded the highest overall test scores. These results were followed by those of the control group, which received instruction through traditional teaching methods. Unlike our study, Linton et al. (27) observed that there were no significant differences in student exam performance between computer-assisted learning and conventional learning methods in canine anatomy. Chen et al. (28) and Canright et al. (29) reported similar findings, observing no significant differences in test performance between students who used VR and 3D models and those who engaged with traditional 2D lectures. Nonetheless, both studies highlighted that students reported greater satisfaction and offered more positive feedback when studying with 3D-based technologies.

The lowest performance was observed in the VR group, characterized by the greatest variability and the widest range of scores. Notably, the VR group’s performance appeared to be significantly influenced by students’ enthusiasm for the immersive and realistic experience of virtual reality. For many participants, it was their first experience with VR technology, which often led to a playful rather than focused approach during the session. We suggest that students’ first contact with technology could have resulted in larger effort to explore the features rather than completely focusing on learning the topic. This reduced the level of attention to instructional content which contributed to their lower test scores. Similar observations were described in a study by Keets et al. (30) where students obtained lower VR exam scores, which may be a result of increased cognitive load associated with using VR. According to Frederiksen et al. (31), immersive VR training in laparoscopy increases cognitive load and negatively affects performance when compared to conventional VR training. Conversely, shorter learning time of the VR group could have yielded lower results since many studies reported the impact of learning time on academic achievement (32). The follow up assessment revealed similar results regarding the score order which were identical with the first test. However, retention rate revealed that VR group demonstrated highest retention of 99%. The results of mixed-ANOVA showed no statistically significant difference between scores considering the group and time of test. However, effect of interaction (*p* = 0.10) indicates the test scores of different learning groups could have different patterns of change. T test showed significant difference between test scores of Control group. This group showed the weakest retention after 15 days, complementing to the evidences that traditional methods of learning are characterized by low retention rate. Since the study was performed on small sample, drawing a definite conclusion is challenging. Thus, the larger groups of students would enhance the statistical power of tests. Nevertheless, our results align with previous research by Zhao et al. (33) who determined that the use of VR in teaching anatomy results in significantly higher post-intervention test scores compared to conventional instructional methods. Similar results were obtained by Huang et al. (34) who provided evidence that individuals who consumed visual and auditory content in VR exhibited greater retention of visual information compared to AR. Another research by Krajčovič et al. (35) conducted in the field of mechanical engineering showed that students who used VR interactive training performed better on tests compared to students who did not use VR. Additionally, students who used VR training demonstrated better knowledge transfer and retention in both short and long-term application. Kadri et al. (36) did research on knowledge retention in VR-based medical education and concluded that collaborative VR learning improves immediate learning of anatomical concepts, as well as enhances the long-term retention of anatomical knowledge compared to non-collaborative VR learning.

The use of VR technology can cause certain discomfort particularly among first-time users. This phenomenon, commonly referred to as cybersickness or VR sickness, is a well-documented side effect (37, 38). The major symptoms of VR sickness are eye fatigue, disorientation, and nausea (39). In our study, the duration of VR exposure was relatively short, and no such symptoms were reported.

To evaluate students’ knowledge, a questionnaire composed of multiple-choice questions was administered. Knowledge of anatomy, like other medical disciplines, is commonly evaluated using a variety of assessment tools, including multiple-choice exams, oral examinations, and objectively structured practical examinations (OSPEs) (40). Among these, multiple-choice questions (MCQs) are a well-established method for assessing both factual knowledge and its clinical application. The key advantage of MCQs is their ease of standardized administration across different institutions and curricula, as well as high levels of reliability and objectivity (41). MCQs were also utilized by previous studies to assess student performance when comparing learning with VR and 3D models to traditional methods (28, 29).

To overcome the limited use of innovative digital tools in identifying meat cuts, we developed high-resolution 3D models by scanning real specimens and integrating them into a virtual reality environment. This resulted in original, immersive educational content. Importantly, the development did not require specialized software, external experts, or advanced technical skills. Instead, it relied on free software solutions and standard digital competencies, highlighting the accessibility and practicality of such approaches in education. The initial investment for the 3D scanner and compatible laptop totaled approximately USD 10,000. To create virtual content, we utilized Open Brush, a free and open-source VR software. An additional cost of USD 1,000 was incurred for the purchase of a VR headset, required to develop the VR-based educational modules.

### Limitations of the study

This study examined the effectiveness of an innovative technological approach in veterinary education, specifically in the training and identification of different meat cuts. However, several limitations should be taken into consideration when interpreting the findings. First, the study involved a relatively small sample size, which limits the generalizability of the results. The target population, fourth-year students at the Veterinary Faculty consists of approximately 35 students, of whom around 60% voluntarily participated in the study. Future research involving a larger and more representative sample would provide more robust and generalizable insights. Additionally, an important indicator of the effectiveness of new educational methods is students’ perception and satisfaction, which should be systematically incorporated into studies evaluating innovative teaching tools. Ultimately, long-term knowledge retention should be evaluated at multiple time points. Furthermore, the study would have benefited from the inclusion of a pre-test to assess participants’ baseline knowledge and to enable comparisons with their performance following the learning sessions.

## Conclusion

The study provides an elaborate description of the preparation of innovative technology-based learning content for the fourth-year students of veterinary medicine. The focus of the study was related to the meat categorization training, along with additional details regarding the common names of the meat cuts, constituent muscles, anatomical locations and the legal classification of the meat cuts. The group of students engaged in self-directed learning using 3D models achieved the best overall test scores, followed by those who received traditional classroom instruction. Although the VR group exhibited lower short-term performance, the results suggest promising outcomes in terms of knowledge retention. Future studies with longer and more structured interventions may yield stronger effects. Integrating technology-based learning tools into veterinary education may improve learning outcomes and represent a valuable complement to traditional instructional approaches.

## Data Availability

The original contributions presented in the study are included in the article/Supplementary material, further inquiries can be directed to the corresponding author.

## Footnotes

^1^ https://sketchfab.com/UNSA-VF
