# Supplementary material for: Anatomy of meat cuts: integrating 3D scanning and virtual reality in veterinary education and training
Source: Front Vet Sci. 2025 Oct 23;12:1680785. doi: 10.3389/fvets.2025.1680785 (PMC12590504; doi:10.3389/fvets.2025.1680785)
Supplement: Supplementary file 1 [file Table_1.docx]

| Group/Variable | Test used | p-value | Normality assumption |
| --- | --- | --- | --- |
| Control group (1. questionnaire) | Shapiro–Wilk | 0.3967 | Normal |
| 3D models group (1. questionnaire) | Shapiro–Wilk | 0.0537 | Normal |
| VR group (1. questionnaire) | Shapiro–Wilk | 0.2159 | Normal |
| Control group (follow-up questionnaire) | Shapiro–Wilk | 0.8500 | Normal |
| 3D models group (follow-up questionnaire) | Shapiro–Wilk | 0.1300 | Normal |
| VR group (follow-up questionnaire) | Shapiro–Wilk | 0.1450 | Normal |

Table 1. Shapiro-Wilk test of normality for each group
