# Supplementary material for: Anatomy of meat cuts: integrating 3D scanning and virtual reality in veterinary education and training
Source: Front Vet Sci. 2025 Oct 23;12:1680785. doi: 10.3389/fvets.2025.1680785 (PMC12590504; doi:10.3389/fvets.2025.1680785)
Supplement: Supplementary file 2 [file Supplementary_file_1.docx]

**GROUP: TRADITIONAL / 3D MODELS / VR SEX: M / F**

1. Which meat cut is shown in the picture?
2. shank
3. tip knuckle
4. flank steak
5. top round


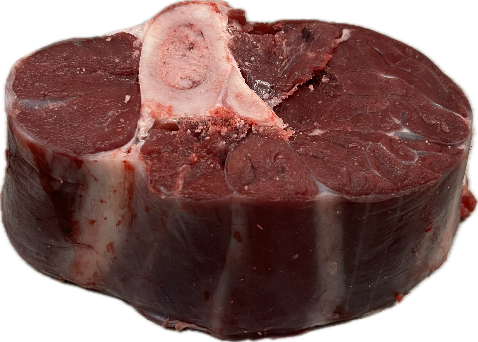


1. *The m. rectus abdominis* and *m. obliquus abdominis externus et internus* make up which cut of meat?
2. tip knuckle
3. flank steak
4. brisket
5. sirloin
6. Which category does the top round belong to?
7. Category I
8. Category II
9. Category III
10. Extra category
11. Which category does the ribeye roll steak belong to?
12. Category I (beef), category II (veal)
13. Category I (veal), category II (beef)
14. Category II (beef), category III (veal)
15. Category III (veal), category II (beef)
16. The brisket consists of the following muscles:
17. *m. rectus abdominis* and *m. obliquus externus et internus*
18. *m. longissimus dorsi*, *m. multifidus*, *m. spinalis*
19. *m. pectoralis profundus et superficialis*
20. Which meat cut is shown in the picture?
21. flank
22. ribeye roll steak
23. top round
24. tip knuckle


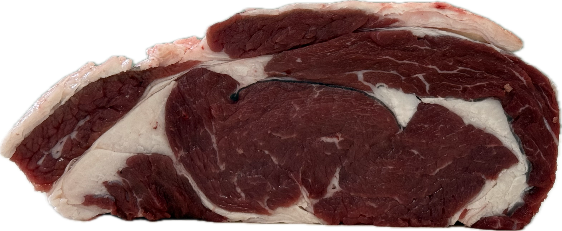


1. Top round is made up of which muscle:
2. *m*. *spinalis*
3. *m*. *semitendinosus*
4. *m*. *semimembranosus*
5. *m*. *psoas* *major*
6. Which category does the whole round belong to?
7. Category I
8. Category II
9. Extra category
10. Which category does the shoulder belong to?
11. Category I
12. Category II
13. Category III
14. The *m. longissimus dorsi, m. multifidus, m. spinalis* and a part of *m.trapezius* make up which cut of meat?
15. sirloin
16. ribeye roll steak
17. tip knuckle
18. whole round
19. Which category does the tenderloin belong to?
20. Category I
21. Category II
22. Trimmings
23. Extra category
24. Which meat cut is shown in the picture?
25. top round
26. whole round
27. sirloin
28. tip knuckle


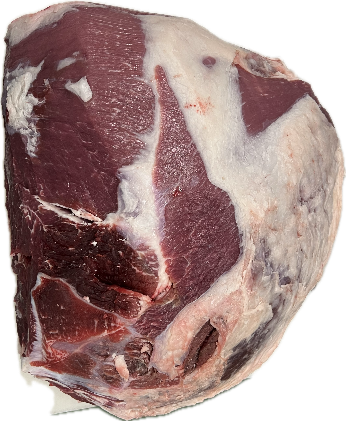


1. Which category does the shank belong to?
2. Category I
3. Category II
4. Category III
5. The beef shoulder is mostly made up of which muscle:
6. *m*. *teres* *major*
7. *m*. *subscapularis*
8. *m*. *supraspinatus*
9. *m*. *infraspinatus*
10. Which meat cut is shown in the picture?
11. top round
12. tenderloin
13. sirloin
14. brisket


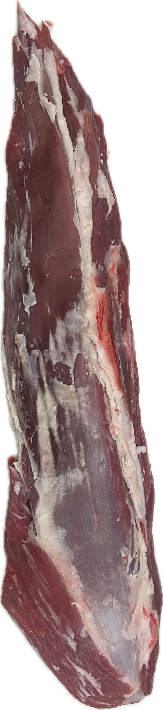


1. Which category does the head meat belong to?
2. Category II
3. CategoryIII
4. Trimmings
5. Extra category
6. Tip knuckle is made up of which muscle:
7. *m*. *biceps femoris*
8. *m*. *quadriceps femoris*
9. *m*. s*emitendinosus*
10. *m*. *semimembranosus*
11. Which meat cut is shown in the picture?
12. brisket
13. tip knuckle
14. shank
15. flank steak


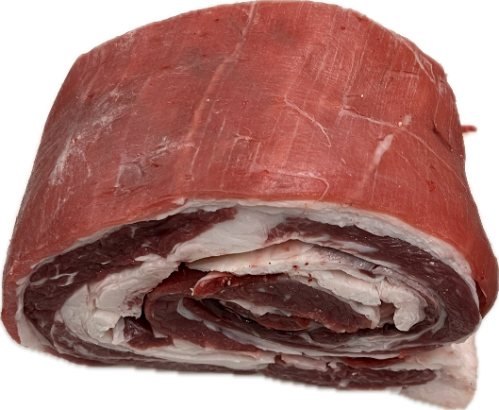


1. Tenderloin is made up of which muscles:
2. *m*. *psoas major et minor*
3. *m*. *longissimus dorsi*
4. *m*. *pectoralis superficialis et profundus*
5. Sirloin represents:
6. musculature located along the spine
7. neck musculature
8. round musculature
9. pectoral musculature
